# Phenotypical divergence between self-reported and clinically ascertained autism

**DOI:** 10.21203/rs.3.rs-4314472/v1

**Published:** 2024-05-07

**Authors:** Sarah Banker, Mathew Schafer, Miles Harrington, Soojung Na, Sarah Barkley, Jadyn Trayvick, Arabella Peters, Abigaël Thinakaran, Jennifer Foss-Feig, Daniela Schiller, Xiaosi Gu

**Affiliations:** Icahn School of Medicine at Mount Sinai; Columbia University Irving Medical Center; Icahn School of Medicine at Mount Sinai; Center for Computational Psychiatry, Icahn School of Medicine at Mount Sinai; Icahn School of Medicine at Mount Sinai; Icahn School of Medicine at Mount Sinai; Icahn School of Medicine at Mount Sinai; Icahn School of Medicine at Mount Sinai; Icahn School of Medicine at Mount Sinai; Icahn School of Medicine, Mount Sinai; Icahn School of Medicine at Mount Sinai

## Abstract

While allowing for rapid recruitment of large samples, online psychiatric and neurodevelopmental research relies heavily on participants’ self-report of neuropsychiatric symptoms, foregoing the rigorous clinical characterization of laboratory settings. Autism spectrum disorder (ASD) research is one example where the clinical validity of such an approach remains elusive. Here, we compared participants characterized online via self-reports against in-person participants evaluated by clinicians. Despite having comparable self-reported autism symptoms, the online high-trait group reported significantly more social anxiety and avoidant behavior than in-person ASD subjects. Within the in-person sample, there was no relationship between self-rated and clinician-rated autism symptoms, suggesting these approaches may capture different aspects of ASD. The online high-trait and in-person ASD participants also differed in their behavior in well-validated social decision-making tasks: the in-person group perceived having less social control and acted less affiliative towards virtual characters. Our study aimed to draw comparisons at three levels: methodological platform (online versus in-person), symptom measurement (self- versus clinician-report), and social behavior. We identified a lack of agreement between self- and clinician-rated measures of symptoms and divergent social tendencies in groups ascertained by each method, highlighting the need for differentiation between in-person versus online samples in autism research.

## Introduction

Online platforms such as Amazon Mechanical Turk (MTurk) and prolific.co have become increasingly popular for data collection in human subject research^1,2^. Through such platforms, researchers can rapidly collect data from hundreds or thousands of participants, allowing for better powered and more diverse samples than traditional laboratory data collection. Despite the many benefits of online research, there are also considerable concerns about the quality and validity of such data^3^. For instance, previous research has reported low test-retest reliability, incoherent answers, and inattention amongst online participants on multiple platforms^3–5^. For studies on psychiatric and neurodevelopmental disorders, current online research primarily relies on self-report surveys to capture symptoms and diagnoses. As many of these conditions are characterized by deficits in insight and/or metacognitive awareness^6–8^, self-report alone may not be the most accurate way to identify individuals with certain diagnoses, or assess objective functioning in certain domains. Without the expert clinical characterizations afforded by lab-based research, there is nothing to compare self-report against, making it difficult to determine its generalizability and ecological or clinical validity.

Such concerns may be especially relevant when studying individuals with autism spectrum disorder (ASD), which is characterized by differences in social- and self-insight in addition to social communication difficulties and behavioral inflexibility. Prior investigations in ASD have highlighted discrepancies between self- and caregiver-reported symptoms of autism^9–11^ and comorbid psychiatric conditions^12^. Though one study conversely identified significant correlations between self- and caregiver-reported autism symptom severity, the combined measures were more predictive of independent living and employment outcomes than either measure alone, suggesting they do not provide redundant information^13^. Additionally, a large-scale study found that self-reported symptoms among adults evaluated for ASD were not predictive of receiving a diagnosis^14^. Such findings suggest that foregoing the collection of outsider reports, as is often done in online research, may impede the contextualization of self-reported symptoms and limit the ability to predict diagnoses and objective outcomes. While self-report provides important information about subjective experience and well-being, it may not be an appropriate diagnostic shortcut.

Discrepancies between self-reported and externally-assessed symptoms in ASD may stem from core socioemotional symptoms, including difficulties with insight and theory of mind (ToM). Impairments in ToM, which refers to the ability to infer the emotions or mental states of others, likely makes it difficult for many individuals with ASD to reliably answer self-assessments inquiring about others’ perceptions of themselves (e.g., the prompt from the Broad Autism Phenotype Questionnaire (BAPQ): “People get frustrated by my unwillingness to bend.”)^15^. Moreover, many individuals with ASD display reduced insight into their internal states^16^. Studies have estimated that somewhere between 50–85% of people with autism experience alexithymia, which is a condition defined by difficulties in identifying, understanding, and expressing one’s own feelings^17,18^. In addition to difficulties in reporting subjective feelings, individuals with ASD may struggle to gage how far their external behaviors deviate from social expectations. When presented with demonstrations of social actions, children and adolescents with ASD are able to successfully identify inappropriate social behaviors (slightly less so for verbalizations) but give abnormal or unrelated reasonings as to why the actions are wrong^19^. This may represent a tendency for people with ASD to internalize social rules using an alternative logic rather than through a true understanding of social norms, a difference that may hinder their ability to assess the extent to which their own actions align with these norms. Thus, impaired insight into others’ perceptions as well as one’s own emotional states and social appropriateness may lead to self-reported symptom levels that differ from those ascertained through outside assessment.

Given such self-assessment biases in ASD, it is likely that self-reported symptoms do not confer the same meaning in individuals with and without a clinical diagnosis. Though sub-clinical autistic traits exist in the general population, psychometric investigations into self-report measures have suggested that equal scores in ASD and non-ASD individuals do not necessarily imply equal levels of autistic traits^20^. In individuals with no ASD diagnosis, a high level of self-reported autistic traits may have little effect on social functioning if they are able to compensate through other adaptive behaviors. In contrast, individuals with ASD may underreport their symptoms due to impaired insight and ToM^9–11^. Furthermore, the relationship between self-reported traits and mental health appear to differ in those with and without a diagnosis; for example, self-reported symptoms are only associated with anxiety in individuals with an ASD diagnosis^21^. Thus, interpretations of self-reported traits in the general population may not be directly applicable to ASD.

In this study, we sought to systematically examine symptom- and socio-cognitive-level similarities and differences between individuals with high autism traits recruited online via self-report and ASD participants defined via in-person clinical characterization. As social impairment is one of the core features of ASD, we chose to compare the online and in-person samples on their behavior during dynamic social interaction tasks. We hypothesized that individuals recruited online who self-reported high-autistic traits would show distinct social interaction tendencies compared to a clinically-defined in-person ASD sample. If true, such results would suggest that self-report alone is not sufficient to identify individuals with ASD in online studies.

## Results

### Autism Symptoms

Participants were enrolled from an online subject pool consisted of “unselected” adults from the community (via Prolific, n = 502) or had diagnoses of ASD confirmed and enrolled for participation in-person (at the Seaver Autism Center in New York City, see Methods for details; n = 56). The online sample was further subdivided into “high-trait” (n = 124) and “low-trait” (n = 121) groups based on their total scores on the Broad Autism Phenotype Questionnaire (BAPQ)^15^. From within each of these groups, 56 age- and sex-matched participants were selected to match the in-person ASD sample. This resulted in three groups with 56 participants each: high-trait, low-trait, and ASD. See Table 1 for demographic characteristics of each group.

**Figure 1:**
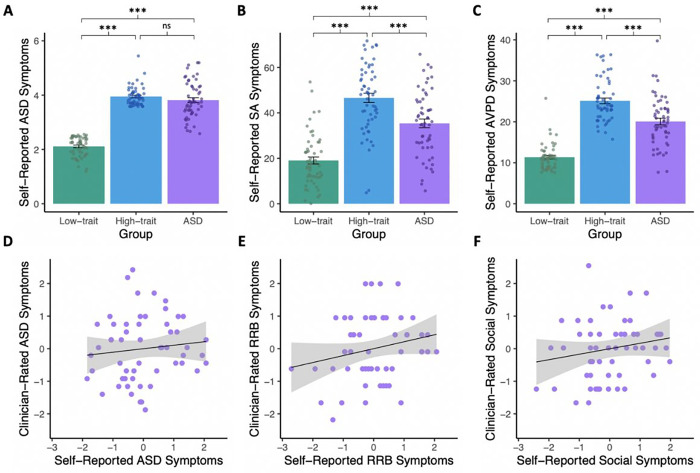
Symptom comparisons. The ASD and high-trait groups have comparable levels of self-reported autism symptoms (measured via BAPQ; t(82.75)=1.31, p=0.32; A). Investigation into symptoms of other disorders characterized social impairment revealed that, compared to both other groups, the high-trait group self-reported a higher level of social anxiety (SA; F(163)= 57.37, p<0.001; B) and avoidant personality disorder symptoms (AVPD; F(163)= 112.68, p<0.001; C). In the in-person ASD sample, there was no relationship between clinician-rated autism symptoms (measured via ADOS) and self-reported autism symptoms (measured via BAPQ; F(51)=0.68, p=0.42; D). Broken down by sub-scales, there was no agreement in the restricted and repetitive behavior domain (RRB; F(51)=2.68, p=0.11; E) or the social domain (F(51)=1.64, p=0.21; F). * p<0.05; ** p<0.01, *** p<0.001

The three groups differed in their self-reported autism symptoms, as measured by BAPQ scores (F(163) = 261.39, p < 0.001, Fig. 1A). As anticipated, this was driven by lower BAPQ scores (indicating fewer symptoms) in the low-trait group compared to both the high-trait group (t(109.95) = 27.67, p < 0.001) and the ASD group (t(83.87) = 16.87, p < 0.001). The high-trait group and the ASD group did not differ in BAPQ scores (t(82.75) = 1.31, p = 0.32). Interestingly, the groups also differed in their social anxiety symptoms (F(163) = 57.37, p < 0.001, Fig. 1B), such that the high-trait group had higher scores (indicating more symptoms) than both the low-trait group (t(102.31) = 10.84, p < 0.001) and the ASD group (t(109.57) = 4.03, p < 0.001), and the ASD group also had higher scores than the low-trait group (t(105.14) = 6.69, p < 0.001). Finally, the groups differed in their avoidant personality disorder symptoms (F(163) = 112.68, p < 0.001, Fig. 1C). The pairwise group differences for avoidant personality disorder symptoms follow the same pattern as social anxiety: the high-trait group had higher scores (indicating more symptoms) than both the low-trait group (t(90.75) = 17.03, p < 0.001) and the ASD group (t(107.55) = 4.75, p < 0.001), and the ASD group also had higher scores than the low-trait group (t(82.90) = 9.62, p < 0.001).

In addition to the self-report measures, in-person participants completed the Autism Diagnostic Observation Schedule (ADOS-2; Module 4)^22^, considered the “gold standard” clinical assessment measure for ASD. Surprisingly, there was no significant relationship between self-reported ASD symptoms measured by BAPQ and those rated by clinicians using ADOS (F(51) = 0.68, p = 0.42, Fig. 1D). Broken down by subdomain, there was also no relationship between self- and clinician-rated symptoms in the restricted and repetitive behavior domain (F(51) = 2.68, p = 0.11, Fig. 1E) or the social domain (F(51) = 1.64, p = 0.21, Fig. 1F). Such limited agreement between self- and clinician-rated assessments suggests that they may not be measuring the same features of ASD: while self-reported assessments can capture subjective internal experiences, clinician-rated assessments may capture external presentation of symptoms. Our results suggest that, in ASD, these two domains do not always agree.

### Social Behavior

As social differences are a hallmark feature of ASD, we chose to compare our groups on their behavior in two dynamic social interactions tasks. The paradigms outlined below allow for the quantification of complex social processes, including exertion of social control and navigation through “social space.”

### Social controllability

**Figure 2:**
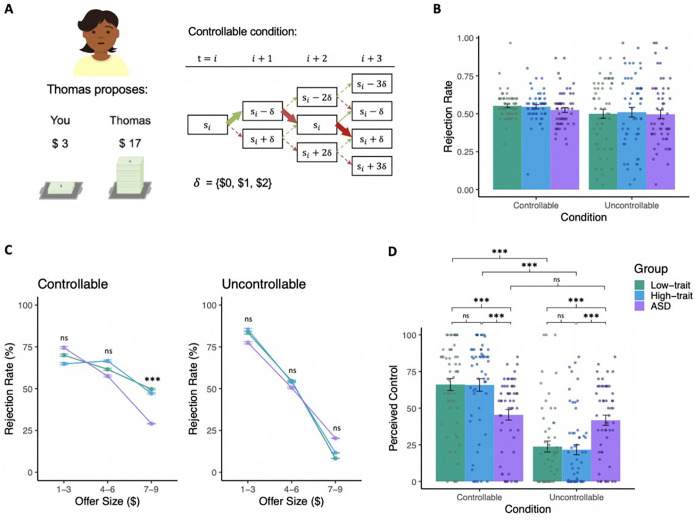
Social controllability task and results. As shown in the representative task screen (A, left), the social control task involved participants accepting or rejecting splits of $20 proposed by members of two virtual teams. Participants played the game with two different teams sequentially, the order of which was counterbalanced. With one of the teams (‘controllable condition’), participants could increase future offers by rejecting the current one, or decrease future offers by accepting the current one (A, right). All groups showed comparable overall rejection rates for both conditions (F(2,328)=0.14, p=0.87; B). When rejection rate is broken down by offer size, we see a group difference in the controllable condition (F(4,355)=2.52, p=0.041) such that the ASD group rejected a higher percentage of high offers than the online groups (F(119)=6.75, p=0.002; C, left); The groups did not differ in rejection rates in the uncontrollable condition (F(4,355)=2.52, p=0.041; C, right). Unlike the online groups, the ASD group did not detect a difference in controllability between the conditions (F(2, 322)=17.77, p=0.001; D). * p<0.05; ** p<0.01, *** p<0.001

Social controllability, or one’s ability to influence other people, is crucial for achieving optimal behavior during dynamic interactions and subsequently, mental wellbeing. To measure social controllability, we used an monetary exchange task^23–26^ modified from the ultimatum game, in which participants decide whether to accept or reject proposed splits of $20 offered by players from two independent teams (Fig. 2A; see **Methods** for details). Unbeknownst to the participants and different from the traditional ultimatum game, participant have control over the offers proposed by one of the teams (“controllable condition”). Specifically, participants can increase future offers by rejecting current ones or decrease future offers by accepting the current ones. At the end of the task, participants rate how much control they believed they had over players from each team (see methods for further task details).

Because participants can only raise offers through rejecting a current proposal, we first sought to characterize their rejection rate, both overall and as a function of offer size. We found that the three groups showed similar overall rejection rates during the task (F(2,328) = 0.14, p = 0.87, Fig. 2B). Breaking rejection rate down by offer size, we found that rejection rate patterns differed by group for the controllable condition (F(4,355) = 2.52, p = 0.041, Fig. 2C). Specifically, though the groups showed similar rejection rates for low and medium offers, the ASD group rejected a smaller percentage of high offers compared to both low- and high-trait online groups (F(119) = 6.75, p = 0.002). Patterns of rejection rates did not differ across groups for the uncontrollable condition (F(4, 493) = 1.94, p = 0.10), with each group showing the highest rejection rates for low offers ($1-$3) and the lowest rejection rates for high offers ($7-$9). Together, these results suggest that high-trait online participants behaved more similarly to the low-trait online group than the clinical ASD group during controllable social interactions, whereas the clinical ASD group demonstrated distinctly reduced ability to exert control.

We next investigated if participants differed in their subjective perception of the controllability they had. Indeed, we detected a significant group-by-condition interaction on perceived control ratings (F(2, 322) = 17.77, p < 0.001, Fig. 2D). In the controllable condition, the ASD group perceived less control than both the high-trait (t(104.36)=−3.63, p = 0.001) and the low-trait groups (t(105.82) = 3.80, p = 0.001); the high- and low-trait groups did not differ from each other (t(109.67)=−0.05, p = 0.99). In the uncontrollable condition, the ASD group reported having more control than both the high-trait (t(106.64) = 4.13, p < 0.001) and the low-trait groups (t(106.85)=−3.52, p = 0.001); the high- and low-trait groups once again did not differ from each other (t(109)=−0.43, p = 0.90). Such results suggest that, compared to both online groups, the clinically-defined ASD sample was less accurate in their ability to detect changes in social controllability. In conjunction with the rejection rate result, these findings suggest that clinically-confirmed ASD individuals, but not those defined solely by high autistic traits, showed altered ability to exert influence and perception of their controllability during social interactions.

### Social Navigation Task

**Figure 3:**
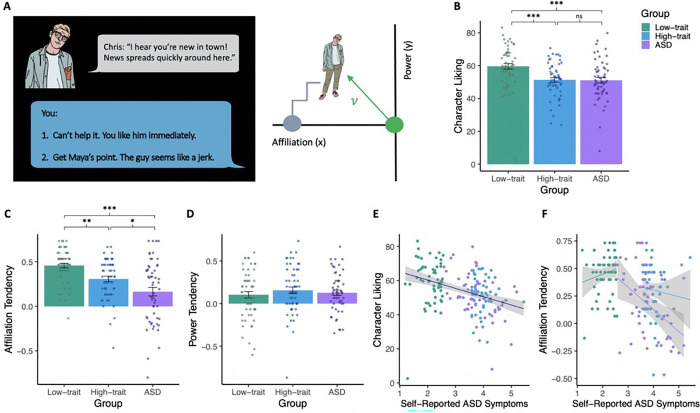
Social navigation. The social navigation task involved participants interacting with different characters with the goal of finding a job and a home. At each interaction, participants could choose between two options that affected either the affiliation or power dynamics of the relationship. Behind the scenes, each decision would move that character’s position accordingly in a “social space” framed by axes of power and affiliation (A). Compared to the low-trait group, the high-trait and ASD groups both reported a reduced liking of the characters in the social navigation task (F(163)=9.04, p<0.001; B). Despite comparable feelings towards characters, the ASD group acted less affiliative than the high-trait group (F(163)=16.97, p<0.001; C). The groups did not differ in their power tendencies (F(163)=0.54, p=0.58; D). There was a negative relationship between character liking and self-reported symptoms across all groups (F(164)=26.80, p<0.001; E). However, the relationship between affiliative behavior and self-reported symptoms differed by group (F(2,160)=34.72, p=0.030); Only the ASD group showed a negative correlation between self-reported symptoms and affiliation tendency (r(54)=−0.38, p=0.01; F). * p<0.05; ** p<0.01, *** p<0.001

The social navigation task^27^ is a narrative-based game in which participants interact with a variety of virtual characters with the goal of finding a job and a place to live (Fig. 3A). The task consists of both story-building narrative trials and choice-point interaction trials. During interaction trials, participants choose between one of two ways to interact with a given character. Unbeknownst to the participant, these choices reflect opposing changes in either the power or affiliation dynamic between them and the characters. At the end of the narrative, participants are asked to rate the characters on how much they liked interacting with them (see **Methods** for further task details).

We began by investigating participants’ subjective feelings towards characters in the task, and found that the three groups differed in their ratings of character likability (F(163) = 9.04, p < 0.001, Fig. 3B). Compared to the low-trait group, both the high-trait (t(106)=−3.72, p = 0.001) and the ASD groups (t(109.91) = 3.55, p < 0.001) self-reported reduced liking of characters. The high-trait and ASD groups did not differ in their character liking (t(107.04)=−0.10, p = 0.99), suggesting comparable subjective experiences.

To explore how each group behaved in the task, we investigated group differences in power and affiliation tendencies, averaged across all characters. A significant 3-group difference in affiliation tendency (F(163) = 16.97, p < 0.001, Fig. 3C) revealed that the ASD group acted significantly less affiliative with the characters than both the high-trait (t(94.04=−2.54, p = 0.014) and low-trait groups (t(85.47) = 5.47, p < 0.001), indicating unique social tendencies in the clinically-defined sample. The high-trait group was also less affiliative than the low-trait group (t(107.29)=−3.79, p = 0.008). The groups did not differ in their power tendencies (F(163) = 0.54, p = 0.58, Fig. 3D).

Last, we explored the relationship between social tendencies and self-reported symptoms or subjective task ratings in each group. There was no group-by-symptom interaction on character liking (F(2,160) = 2.50, p = 0.085). Rather, across all groups, there was a significant negative relationship between character liking and self-reported symptoms (F(164) = 26.80, p < 0.001, Fig. 3E), indicating that those with higher self-reported autism symptoms liked the characters less. Finally, there was a significant group-by-symptom interaction on affiliation tendency (F(2,160) = 34.72, p = 0.030, Fig. 3F). While the ASD group showed a negative correlation between self-reported symptoms and affiliation tendency (r(54)=−0.38, p = 0.01), there was no relationship in the high-trait (r(54)=−0.09, p = 0.50) or low-trait groups (r(54) = 0.17, p = 0.23). Thus, while the relationship between subjective ratings and self-reported symptoms did not differ by group membership, the relationship between objective behavior ratings and self-reported symptoms was specific to the clinical sample.

## Discussion

In the current study, we sought to investigate the phenotypic similarities and differences between online participants with high self-reported autism traits and those with an ASD diagnosis confirmed in-person via clinician evaluation. We identified a lack of agreement between self-rated and clinician-assessed symptom measures, highlighting the need for separate interpretations of each. When investigating each group’s social behavior, we found that individuals with confirmed ASD showed impairments in recognizing opportunities to exert social control and reduced affiliation in their interactions with virtual characters; in contrast, high-trait individuals identified online showed comparable social behaviors to low-trait individuals. These results provide a caution for future online research: when attempting to identify and draw conclusions about certain diagnostic groups, self-report alone may not be sufficient.

Despite the overall lack of measurement agreement identified in this study, we do not believe that these results suggest that self-report questionnaires are invalid for ASD research. On the contrary, they are important tools for understanding individual’s subjective experiences and levels of internal distress or wellbeing. Self-reports are also critical for ensuring individuals with lived experience have a role in shaping the narrative surrounding them, and can help challenge baseless assumptions regarding the intentions or reasoning behind autistic behavior. Rather than dismiss the importance of subjective self-views, the results provide a caution for the use of self-report alone for defining or extrapolating about a diagnostic group.

We saw no relationship between self-reported BAPQ and clinician-rated ADOS symptom scores in the in-person ASD group, consistent with previous reports using different measures^9–11^. Discrepancies between self- and observer-rated symptoms are not uncommon amongst individuals with altered introspection; they have been reported in a variety of conditions characterized by impaired insight, including depression^28^ and schizophrenia^29^. Evidence suggests that insight difficulties in such conditions may be more pronounced in certain domains. Among individuals with schizophrenia, for instance, those with reduced insight have been shown to over-report their levels of extroversion but accurately reported other personality traits, suggesting insight may play a significant role in the reporting of social tendencies specifically^30^. Reduced social self-insight has been widely reported in ASD^31,32^ and likely contributes to discrepancies between self- and clinician-report. It is possible that, despite presenting with relatively normative social behavior to the outside observer, the ASD individuals with higher social awareness report experiencing more social difficulties due to increased insight into their social limitations and differences from typically developing peers^31,33^.

In the social controllability task, the ASD group rejected a smaller percentage of high offers in the controllable condition compared to the online groups. This reduced rejection of “good” offers hindered their ability to receive better offers down the line, suggesting they did not take advantage of the controllability offered by the condition. In line with this, we also saw that the ASD group did not self-report any differences in the perceived controllability of the conditions. Such results may stem from reductions in ToM-related understanding of others’ motivations in the clinical ASD group but not the high-trait group. To distinguish between random and non-random behaviors on the part of the players, one must realize that they are motivated to receive the largest amount of money possible. To achieve this understanding, you might use prior information (i.e., past offers) to build expectations about future behaviors (i.e., players will give you repeatedly low offers as long as you continue to accept them) that would fit a given intention (i.e., players want to maximize gain) and evaluate their accuracy. In ASD, impaired ability to predict offers and understand players’ intentions may lead to a lack of distinction between random and non-random (goal-directed) behavior. Indeed, individuals with ASD display reduced understanding of social intentions, including whether actions are goal-directed^34^, that appears to stem from impaired use of prior social information to form expectations^35^. It’s also possible that the reduced perception of controllability seen in ASD is caused by impaired affordance perception, which refers to the ability to ascertain which actions are available for you to take in a given environment. Autistic individuals have been shown to inaccurately estimate action capabilities in the perceptual-motor domain^36^, and such impairments are theorized to extend into the social domain^37^. In any case, the high-trait and low-trait online groups showed comparable behavior across all task measures, suggesting that this impaired detection of others’ goal-directed behaviors and/or perception of the actions available to oneself is specific to individuals with a confirmed ASD diagnosis.

In the social navigation task, though both the high-trait and ASD groups reported liking the characters less than the low-trait group, only the ASD group was less affiliative with characters during their interactions than other groups. Such results highlight the importance of measuring behavior for achieving a comprehensive understanding of symptom presentation. The high-trait and ASD groups were aligned in their subjective beliefs, both about their symptoms and their opinions of others, but these beliefs did not translate into comparable social tendencies. Considering that pro-affiliative behavior is often considered to be polite, and that individuals with ASD frequently exhibit diminished adherence to social conventions^38^, this difference may be reflective of reduced awareness of or desire to follow friendliness norms in ASD. In contrast, those without a confirmed diagnosis may be more inclined or better able to act friendly despite their internal discomfort and dislike of characters. In line with this idea, though reduced character liking was associated with increased self-reported symptoms in all groups, we only detected a relationship between self-reported symptoms and affiliative behavior within the ASD group – those with a higher level of symptoms were the least friendly with the characters. Such results provide further evidence that self-reported symptoms have difference implications in individuals with and without a confirmed ASD diagnosis. Altogether, the findings from both tasks suggest that samples defined by online self-report are phenotypically distinct from clinically-ascertained samples, and that using such online samples to answer questions about social interaction may not be informative about ASD as a whole.

In our study, the online group with high autistic traits also self-reported heightened levels of social anxiety and avoidant personality disorder symptoms compared to the in-person ASD group. This difference suggests that self-reported autism symptoms in the general population may be more reflective of general social avoidance and self-consciousness regarding social skills rather than autism-specific social difficulties. Supporting the existence of this phenotype, largescale online studies investigating latent psychiatric factors in the general population have identified transdiagnostic dimensions characterized by similar socially-avoidant/anxious traits^39,24^. As we have shown, these online participants who report elevated internal social difficulties (i.e., emotional or cognitive struggles that others may not notice, as described by self-report) also show different social behaviors from those with a clinical diagnosis who show elevated external difficulties (i.e., inappropriate actions or visible struggles, as described by clinician-report), suggesting the diagnosis and the dimension are not synonymous. Though ASD is highly comorbid with social anxiety, it is still only represented in less than half of cases^40^, and comorbidity with avoidant personality disorder is even less common^41^. It may be the case that self-reported internal symptoms lack diagnostic specificity, especially at subclinical thresholds, whereas clinicians are able to better assign clinically-significant symptoms to separate diagnoses through observing external behaviors.

An important implication of this distinction is that we must be cautious not to extrapolate about the needs of one group based on the findings from research conducted in the other. For example, the individuals with high self-reported autism, anxiety, and avoidance traits, despite doing reasonably well by external metrics of social abilities, may need intervention towards boosting self-confidence, and reducing anxiety and negative self-talk rather than social skills trainings. In contrast, the individuals who self-report few symptoms but present to clinicians with observable difficulties in social interaction may benefit from more skills-focused training to aid in quality-of-life outcomes like independent living, relationships, and employment. This distinction is important because it presents a potential risk of harm (or at least reduced access to benefits) to autistic individuals who require more behavioral support and their access to accommodations; If online self-report-based samples are used to represent the whole diagnostic spectrum despite clear differences in behavior, the implications for intervention may be biased.

This study should be interpreted with the following limitations in mind. First, we relied on a single self-reported autism symptom measure - the BAPQ - because of its strong psychometric properties in both the general population and in those with an ASD diagnosis^15,42,43^. However, other surveys such as the Autism-Spectrum Quotient (AQ)^44^ are also commonly used in research to assess autistic traits and do not always converge with clinical/caregiver impressions^9,12,14^, similar to the BAPQ-ADOS discrepancy identified in the current study. Second, since the inception of this study, Prolific has added a screening tool that allows researchers to specifically select participants that self-report having received a formal clinical diagnosis of ASD. However, this information is still self-reported and unverifiable. Future work should investigate if the use of additional symptom measures and/or self-reported diagnoses in online studies would identify a group that shows behavior more closely aligned with the ASD phenotype. Lastly, we do not have evidence to examine if the current findings are specific to ASD or generalizable to other psychiatric diagnoses such as schizophrenia or personality disorders where impaired insight can be a symptom. Future research is needed to investigate the broader implications of this work.

As online research continues to proliferate, we must consider the limitations of online approaches when determining which scientific questions they are best suited to answer. Questions about transdiagnostic traits and symptoms, for example, avoid the issues with diagnostic specificity in self-report and may be well-suited for testing with online platforms, especially for traits not associated with impaired insight. Online research is a powerful tool that will continue to help answer important questions in human-subject research. However, the results of the current study suggest that online approaches in psychiatry should be used in tandem with, rather than as a replacement for, lab-based research, and that over-generalization of findings should be avoided in research relying on self-reported symptoms. For questions that require big-data, researchers have other tools at their disposal: pooling resources, developing cross-site collaborations, or utilizing resources like Simons Foundation Powering Autism Research (SPARK)^45^ will allow for large-scale replications of lab-based studies in ASD that are less reliant on self-report.

## Materials and Methods

### Participants

The study was approved by the Institutional Review Board at the Icahn School of Medicine at Mount Sinai and all participants provided informed consent prior to participation. The authors assert that all procedures contributing to this work comply with the ethical standards of the relevant national and institutional committees on human experimentation and with the Helsinki Declaration of 1975, as revised in 2008.

Online participants were enrolled in the study as part of a larger online project examining social cognition and mental health. Participants were recruited from Prolific (www.prolific.co), an online research participant recruitment site, with the eligibility criteria of (1) age between 18 and 64, (2) currently living in the United States, (3) > 90% approval rating in Prolific. Participants provided consent by clicking “I Consent” after reading information about the study and were paid for their participation after completion, in accordance with policies on Prolific and at Mount Sinai’s School of Medicine. A total of 1,499 individuals attempted the initial study, which included the social controllability task (April 2020). From this, 14 participants were excluded due to duplication of their data files and an additional 143 participants were excluded for flat behavior during the task (accepting or rejecting all offers). Of the initial push, 1,269 responded to a follow-up study containing relevant questionnaires (June - August 2020); 38 were excluded for questionnaire non-completion, 9 were excluded for exceeding the questionnaire time limit, and 47 were excluded due to missed attention checks or ID errors. This resulted in a total of 1,041 participants with usable questionnaire and social controllability task data. Also out of the initial push, 733 participants responded to a follow-up study to complete the social navigation task (April 2021 - January 2022); 157 were excluded for either not having a plausible average decision response time (within +/− 2 standard deviations of mean) or having at- or below-chance post-task memory, resulting in 576 participants with complete social navigation task data. In total, 502 online participants completed all aspects of the study.

Eighty-eight in-person participants enrolled in the current study and were screened for inclusion/exclusion by clinical staff at the Seaver Autism Center for Research and Treatment at the Icahn School of Medicine at Mount Sinai (August 2021 - June 2023). Participants were recruited through announcements posted on physical flyers around New York City and email listservs with the eligibility criteria of (1) age between 18 and 50, (2) meet criteria for ASD, (3) IQ > 70. Participants were screened for ASD by licensed, research reliable clinicians with the ADOS-2, and as-needed with developmental and clinical history. Of the 88 initially enrolled in the study, 4 were excluded due to a loss to follow-up and/or unavailability to come into the lab. Of the 84 who attempted the tasks, 64 performed the tasks inside of the MRI to examine neural questions for an additional study, and 20 performed the tasks outside if the scanner on a laptop due to MRI contraindications. Both groups were included in this study. To be included in the final sample for the social navigation task, participants had to respond on at least 75% of trials and have above chance post-task memory scores. To be included in the final sample for the social controllability task, participants could not have flat behavior (e.g., rejected or rejected all offers in either condition). After exclusion, the final sample for the social navigation task was 71 participants and the final sample for the social controllability task was 67 participants; 56 successfully completed both tasks without exclusion.

### Measures

To assess levels of autistic traits in the sample, all participants completed the Broad Autism Spectrum Questionnaire (BAPQ; Hurley et al., 2007). The BAPQ was selected due to its high sensitivity and specificity and good test-rest reliability^15,42,43^. It is worth noting that the BAPQ was originally designed to assess autistic traits in the non-autistic relatives of individuals with ASD, and that the validity of BAPQ in clinical populations has been debated^46,47^. However, its strong psychometric properties^15,42^ and lack of ceiling effects^48^ in individuals with ASD suggest that it performs well in clinical populations^43^ as well as the general population. All participants completed additional questionnaires to investigate symptoms of other psychiatric diagnoses, including Liebowitz Social Anxiety Scale (LSAS Avoidance questions; Liebowitz, 1987) and the Avoidant Personality Disorder Impairment Scale (AvPD-IS; Liggett et al., 2017). The in-person participants additionally completed the Autism Diagnostic Observation Schedule (ADOS-2; Module 4)^22^, a standard clinical assessment measure for ASD.

### Grouping

The full online sample (n = 502) was subdivided into those who scored in the top 15% on BAPQ scores (“high-trait”, n = 124) and those who scored the bottom 15% on BAPQ scores (“low-trait”, n = 121). To minimize potential differences between in-person and online samples, we selected age- and sex-matched participants from within both high- and low-trait online groups to match the in-person ASD group. This resulted in three groups with 56 participants each: high-trait, low-trait, and ASD.

## Experimental paradigms

### Social controllability task

The social controllability task^23–25^ investigates how individuals exploit control over others to maximize rewards. Participants were paired with virtual players from two 30-person teams, denoted by a town name (“Aldertown” and “Banyan Bay”) as well as a common color for the background and team members’ shirts. In each trial, the virtual partner proposed way to split $20 (e.g., $8 for you, $12 for them), and the participant had to decide whether to accept or reject the offer. If the participant chose to accept, both parties received the proposed amounts. If they chose to reject the proposal, neither party received any money. Each team represented a different condition: Controllable or Uncontrollable. Though the participants were told that they “may or may not have influence over this team’s offers,” they were not explicitly instructed that they had control over only one team, or which team represented which condition. The order of the conditions was randomized across participants. Importantly, a prior study using this task showed clear differences when participants were instructed that they were ‘playing with computer’ instead of ‘playing with virtual human partners,’ suggesting the human version of the task successfully probes social-specific behaviors^23^.

In the Controllable condition, participants could either increase the value of the next offer by rejecting the current offer or decrease the value if the next offer by accepting the current offer. The amount of the offer change was determined in a probabilistic manner: chance of changing the offer by $2, chance of changing the offer by $1, and chance of no change. In contrast, in the Uncontrollable condition, offer amounts were randomly sampled from a predetermined distribution (mean = $5.0, SD = $2.3) and the order of task conditions was randomized for each participant. In both conditions, the initial offer was $5 and the offers were constrained to be an integer between $1 and $9 (inclusive). At the end of the task, participants were asked to rate how much control they perceived they had over each team on a scale of 0–100%.

### Social Navigation Task

The social navigation task^27^ is a narrative-based game in which participants interacted with a variety of virtual characters. To adapt the original task for use in a clinical population and allow added check-ins as needed, the task was divided into 4 runs of roughly equal length, following the natural cut points in the narrative (i.e., transition into a new scene). At the start of the game, participants were told they had just moved to a new town and needed to find a job and a place to live. They were asked not to overthink their choices and to behave as they would in real life. The task consisted of narrative trials, which contained images of characters and narrative-progressing text, and decision trials, in which the participant had to choose between two ways of interacting with a given character. To select a choice, participants pressed key 1 or 2 on the in-scanner button box. Though the task appeared to follow a “choose your own adventure” style of dynamic storytelling, the slides were actually the same regardless of participants’ decisions. The slides that appeared after the decision trials were written to have narrative continuity regardless of the specific decisions that were made. To minimize the potential for internal biases influencing results, the race (light- vs dark-skinned) and gender (masculine vs feminine presenting) of the characters were counterbalanced (for in-person participants) or randomized (for online participants) across versions. After the task, participants completed a set of questions, including ratings of how much they liked the characters, as well as a set of memory questions to ensure attention during the task.

Unbeknownst to the participant, each decision trial in the task probed choices in either the affiliation or power domain. Affiliation decisions included, for example, whether or not to share physical touch, physical space, or information (e.g., to share their thoughts on a topic). Power decisions included, for example, whether to submit to versus issue a directive/command, or otherwise exert versus give control. Each option would lead to changes in opposing directions, coded as either + 1 or −1 depending on whether it was pro- vs anti-affiliative for the affiliation trials, or gave power to the character vs took power away from the character for the power trials. The order of the options within a decision trial was counter-balanced across participants. Over the course of the narrative, participants interacted with 5 different characters holding a variety of social roles, each with 6 affiliation and 6 power decisions, for a total of 60 decisions. There was also a neutral character with 3 neutral decisions that did not change their social location; these trials were not included in these analyses.

Behind the scenes, participants’ choices during the decision trials moved the characters positions within a 2D “social space” framed by axes of power and affiliation. Each character started at the origin, with neutral affiliation and power (0,0). With each decision, that character’s coordinates were updated in the positive or negative direction along the current dimension. If, for instance, the participant chose the pro-affiliative option in an affiliation decision trial, that character would move one unit in the positive direction on the affiliation axis. Thus, at any point in the task, the characters’ 2D coordinates were the cumulative sums of the participant’s affiliation and power decisions in those specific relationships. To get summary measures of participants’ social tendencies, we calculated the means of their decisions in the power and affiliation domains separately for each character, and then averaged across characters.

### Statistics

To test for agreement between self-rated and clinician-rated autism symptoms, regression models investigated the relationship between z-scored BAPQ scores and z-scored ADOS scores in the in-person sample. As an exploratory follow-up, we also tested for relationships between corresponding normed subdomains (restricted and repetitive behaviors: BAPQ “Rigid” subscale and ADOS “Restricted and Repetitive Behaviors” subdomain; social behavior: averaged BAPQ “Aloof” and “Pragmatic Language” subscale and ADOS “Social Affect” subdomain, all z-scored). All statistical tests controlled for age and sex.

To investigate differences in self-reported autism symptoms, ANOVAs tested for differences in BAPQ scores across all 3 groups. As an exploratory follow-up to further characterize the groups, we also used ANOVA to test for differences in symptoms of other psychiatric disorders characterized by differences in social behavior: avoidant personality disorder and social anxiety. Significant 3-group ANOVAs were followed up by Welch’s t-tests to parse the direction of the effects, following Tukey’s procedure to adjust p-values for multiple comparisons.

In the social controllability task, two-way ANOVAs investigated group-by-condition interactions on overall rejection rates and perceived control. For rejection rate patterns, two-way ANOVAs investigated group-by-offer size interactions on rejection rate in each condition separately. One-way ANOVAs investigated group differences in behavior in the social navigation task. We specifically investigated differences in power and affiliation behavioral tendencies, as well as ratings of how much they liked the characters. Significant ANOVAs were followed up by Welch’s t-tests to parse the direction of the effects, following Tukey’s procedure to adjust p-values for multiple comparisons.

Finally, to investigate whether the relationship between symptoms (e.g., BAPQ scores) or subjective task ratings/social behavior differed as a function of group, we used 2-way ANOVAs to test for group-by-symptom and group-by-rating interactions on social navigation task variables and 3-way ANOVAs to test for group-by-condition-by symptom interactions on social control task variables. Non-significant interaction terms were dropped from the models to investigate main effects across all groups. To understand the individual relationships, significant interactions were followed up by Pearson’s correlations in each group, corrected for multiple comparisons by hypothesis using the Benjamini–Hochberg method. All statistical tests reported in this study controlled for age and sex.

## Figures and Tables

**Table 1:** Group demographic information. Demographic information is shown for the ASD, high-autistic trait, and low-autistic trait groups. Welch t-tests examined differences between continuous variables, one-way ANOVA examined differences between factor variables. For race and ethnicity, counts and percentages for the most common category are shown. ns = not significant.

	ASD (n = 56)	High-trait (n = 56)	Low-trait (n = 56)	3-group difference
Aged mean (sd)	28.07 (8.53)	28.20 (6.52)	28.73 (6.21)	ns
Sex % Female (n)	51.8% (n = 29)	51.8% (n = 29)	51.8% (n = 29)	ns
Race % White (n)	69.6% (n = 39)	69.6% (n = 39)	69.6% (n = 39)	ns
Ethnicity % Hispanic/Latino (n)	23.2% (n = 13)	10.7% (n = 6)	16.1% (n = 9)	ns

## Data Availability

Data and code for this study is available at https://github.com/smbneuro5/Online_Vs_InPerson_AutismResearch/tree/main
